# Intraperitoneal Granulomas Unexpectedly Found during a Cesarean Delivery: A Late Complication of Dropped Gallstones

**DOI:** 10.1155/2017/4873273

**Published:** 2017-11-23

**Authors:** David A. Suarez-Zamora, Luis E. Barrera-Herrera, Ricardo Caceres-Mileo, Mauricio A. Palau-Lazaro

**Affiliations:** ^1^Department of Pathology and Laboratories, Fundación Santa Fe de Bogotá, Bogotá D.C., Colombia; ^2^Department of Obstetrics and Gynecology, Fundación Santa Fe de Bogotá, Bogotá D.C., Colombia; ^3^School of Medicine, Universidad de los Andes, Bogotá D.C., Colombia

## Abstract

Laparoscopic cholecystectomy is the treatment of choice for patients with symptomatic cholelithiasis. Spillage of gallstones into the abdominal cavity during laparoscopic cholecystectomy occurs in approximately one-third of cases. Although retained gallstones remain asymptomatic, few cases may develop complications. We report the case of a 29-year-old nulliparous woman presenting with several hard nodules in the omentum, raising the possibility of a metastatic disease. Histological examination demonstrated a bile-stained material and a foreign body-type granulomatous response without neoplastic tissue. Our case demonstrates an example of a complication resulting two years after a laparoscopic cholecystectomy that was unexpectedly found during a cesarean delivery. Pathologists should be aware of this entity to avoid interpretation errors.

## 1. Introduction

Laparoscopic cholecystectomy (LC) has become the treatment of choice for patients with symptomatic cholelithiasis [1]. A common complication of this surgical procedure is the gallbladder perforation and spillage of gallstones into the abdominal cavity [2], which occurs in approximately 30% of cases [3]. Studies suggest that 16 to 50% of dropped stones remain unrecovered [4]. Although most of the retained abdominal gallstones remain clinically silent [5], it is estimated that 8.5% of these stones can result in complications [3]. Diagnosis is challenging due to atypical clinical presentation, inability to visualize stones with conventional radiological imaging, and unexpected locations of dropped gallstones [6]. We describe a rare case of intraperitoneal granulomas due to spilled gallstones that were unexpectedly found during a cesarean delivery.

## 2. Case Presentation

A 29-year-old nulliparous woman was admitted at 40 weeks of gestation for labor induction. A cesarean delivery was performed due to failure to respond to induction with dinoprostone and misoprostol. During the surgical procedure, several hard nodules in the omentum were unexpectedly found. The ovaries and other abdominal organs appeared normal. Twenty nodules were carefully dissected and removed. There was a concern that these lesions may represent metastases from an unknown primary tumor. Therefore, the nodules were sent for histopathological examination. Recovery was uneventful and the patient was discharged on her 2nd postoperative day. A LC two years prior with spillage of gallstones during the procedure was documented in the patient's record. Ultrasound images were not available at our institution.

**Figure 1:**
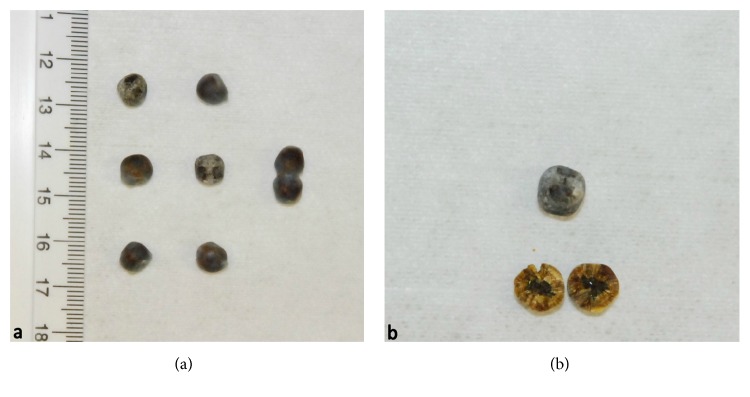
Macroscopic appearance of nodules collected from the patient, resembling gallstones of cholesterol.

**Figure 2:**
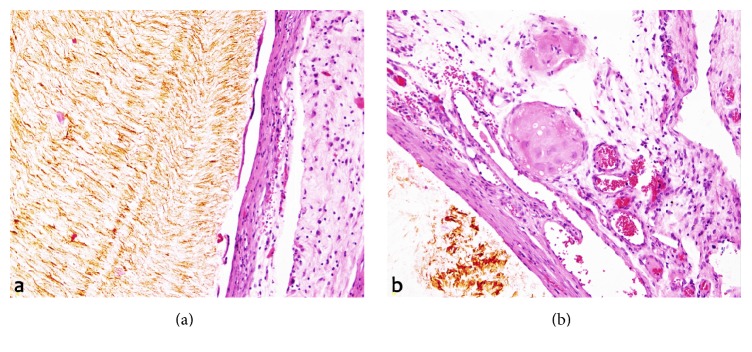
(a) H&E-20x, fibrous capsule surrounding the gallstone. (b) H&E-20x, occasional multinucleated foreign body giant cells without evidence of neoplastic tissue.

Grossly, the specimen consisted of several brownish-gray nodules within the omentum measuring up to 6 mm in diameter with a cut surface closely resembling gallstones of cholesterol (Figure 1). Routine histological examination showed that the nodules were well circumscribed by a fine fibrous capsule. A bile-stained material surrounded by a foreign body-type granulomatous response was noted and there was no evidence of neoplastic tissue (Figure 2). These findings were consistent with the diagnosis of granulomatous peritonitis after LC.

## 3. Discussion

Since its initial description in 1985 by Erich Mühe, the LC has become the treatment of choice for patients with symptomatic cholelithiasis [7]. The increasing use of this laparoscopic procedure has resulted in an increased incidence of spillage of gallstones into the abdominal cavity, which occurs in approximately 30% of cases [5] and mostly happens during the dissection of the gallbladder or during the removal of the organ through port sites [1, 3].

Although most of the retained abdominal gallstones remain asymptomatic [5], it is estimated that 8.5% of these unretrieved gallstones may cause a variety of complications [3]. The most common complications related to lost gallstones after laparoscopy include infection, inflammation, fibrosis, adhesion, bowel obstruction, and fistula and intra-abdominal abscess formation [8]. To the best of our knowledge, only two cases of intraperitoneal granulomas as a result of retained gallstones that were unexpectedly found during a cesarean delivery have been previously reported in the literature [9, 10].

These peritoneal granulomatous nodules may be misinterpreted as peritoneal metastases, focal liver masses, and lymph nodes [6]. In our case, there was a clinical concern regarding an occult disseminated malignancy, but histopathological findings of deposits of bile-stained material, surrounded by a granulomatous response and history of LC two years prior, supported the diagnosis of granulomatous peritonitis as a result of dropped gallstones.

Other unusual causes of granulomatous peritonitis include tuberculosis, sarcoidosis, Crohn's disease, Wegener's granulomatosis, lymphoproliferative disorders, and foreign resources such as talc and barium, meconium, intestinal secretions, contents of ruptured ovarian cysts, and even bile acids [11, 12]. Some authors have suggested that peritoneal exposure to bile salts may cause a foreign body-type granulomatous reaction in certain patients [11]. During pregnancy, potential causes of intraperitoneal nodules such as ectopic decidua, endometriosis, and disseminated peritoneal leiomyomatosis should be included in the differential diagnosis [10]. According to this case and the increasing use of LC, it is important to consider nodules in the abdomen due to spilled gallstones as a differential diagnosis of intraperitoneal granulomas.

To avoid this type of complication during LC, removal of the intact gallbladder is the best strategy to prevent retention of dropped gallstones in the abdomen [3]. If gallbladder perforation occurs, the careful removal of as many gallstones as possible is recommended to reduce the incidence of complications [13]. When the spilled stones are not retrieved completely, intense irrigation of the surgical area together with the use of suction devices should be performed in order to minimize the number of lost gallstones [14].

In summary, some patients with retained gallstones in the abdominal cavity may develop complications. This is the second case of intraperitoneal granulomas after LC that was unexpectedly found during a cesarean delivery mimicking metastases. It is mandatory to highlight the importance of removing as many gallstones as possible in order to reduce future complications. Pathologists should be aware of this entity to avoid interpretation errors.
